# Prophylactic Zinc and Therapeutic Selenium Administration Increases the Antioxidant Enzyme Activity in the Rat Temporoparietal Cortex and Improves Memory after a Transient Hypoxia-Ischemia

**DOI:** 10.1155/2018/9416432

**Published:** 2018-09-06

**Authors:** Constantino Tomas-Sanchez, Victor-Manuel Blanco-Alvarez, Daniel Martinez-Fong, Juan-Antonio Gonzalez-Barrios, Alejandro Gonzalez-Vazquez, Ana-Karina Aguilar-Peralta, Maricela Torres-Soto, Guadalupe Soto-Rodriguez, Ilhuicamina Daniel Limón, Eduardo Brambila, Lourdes Millán-Pérez-Peña, Jorge Cebada, Carlos E. Orozco-Barrios, Bertha Alicia Leon-Chavez

**Affiliations:** ^1^Facultad de Ciencias Químicas, Benemérita Universidad Autónoma de Puebla, 14 sur y Av. San Claudio, 72570 Puebla, PUE, Mexico; ^2^Departamento de Fisiología, Biofísica y Neurociencias, Centro de Investigación y de Estudios Avanzados del Instituto Politécnico Nacional, Apartado Postal 14-740, 07000 Mexico City, Mexico; ^3^Laboratorio de Medicina Genómica, Hospital Regional 1° de Octubre, ISSSTE, Avenida Instituto Politécnico Nacional No. 1669, 07760 Mexico City, Mexico; ^4^Facultad de Medicina, Benémerita Universidad Autónoma de Puebla, 13 sur 2702, Los Volcanes, 72420 Puebla, PUE, Mexico; ^5^Centro de Química, ICUAP, Benémerita Universidad Autónoma de Puebla, 14 sur y Av. San Claudio, 72570 Puebla, PUE, Mexico

## Abstract

In the cerebral hypoxia-ischemia rat model, the prophylactic administration of zinc can cause either cytotoxicity or preconditioning effect, whereas the therapeutic administration of selenium decreases the ischemic damage. Herein, we aimed to explore whether supplementation of low doses of prophylactic zinc and therapeutic selenium could protect from a transient hypoxic-ischemic event. We administrated zinc (0.2 mg/kg of body weight; ip) daily for 14 days before a 10 min common carotid artery occlusion (CCAO). After CCAO, we administrated sodium selenite (6 *μ*g/kg of body weight; ip) daily for 7 days. In the temporoparietal cerebral cortex, we determined nitrites by the Griess method and lipid peroxidation by the Gerard-Monnier assay. qPCR was used to measure mRNA of nitric oxide synthases, antioxidant enzymes, chemokines, and their receptors. We measured the enzymatic activity of SOD and GPx and protein levels of chemokines and their receptors by ELISA. We evaluated long-term memory using the Morris-Water maze test. Our results showed that prophylactic administration of zinc caused a preconditioning effect, decreasing nitrosative/oxidative stress and increasing GPx and SOD expression and activity, as well as eNOS expression. The therapeutic administration of selenium maintained this preconditioning effect up to the late phase of hypoxia-ischemia. Ccl2, Ccr2, Cxcl12, and Cxcr4 were upregulated, and long-term memory was improved. Pyknotic cells were decreased suggesting prevention of neuronal cell death. Our results show that the prophylactic zinc and therapeutic selenium administration induces effective neuroprotection in the early and late phases after CCAO.

## 1. Introduction

Zinc plays a dual role in the cerebral hypoxia-ischemia depending on its concentration in the cerebral stroke area; this concentration is known to be determined by zinc serum levels [1]. Accordingly, low serum levels of zinc have long been considered as a risk factor for stroke [2]. In contrast, the input of low concentration of zinc chloride (ZnCl_2_) or zinc protoporphyrin (ZnPP) reduces the size of postischemic brain damage [3]. Several mechanisms can be accounted for the latter effect. For instance, the decrease in interleukin-1 (IL-1) and IL-23 expression [4], increase in chemokine and growth factor levels [5], and decrease in oxidative stress are because of the antioxidant activity of Cu and Zn superoxide dismutase (SOD1 and 3) [6]. However, excessive accumulation of zinc can also cause neuronal degeneration in the hippocampus and cerebral cortex [7]. Therefore, hypothermia by preventing the accumulation of zinc decreases the cell death [8–10], the degeneration of hippocampal neurons, and the loss of memory after hypoxia-ischemia [11]. The mechanism underpinning the hypothermia effect is the reduction of zinc transport from the presynaptic neurons into the postsynaptic neurons during experimental global ischemia.

Selenium has been shown to preserve mitochondrial function, stimulate mitochondrial biogenesis, and reduce infarct volume after focal cerebral ischemia [12]. Selenium treatment in a rat ischemia model decreases oxidative stress [13]. Furthermore, the oral administration of selenium improves learning and memory in an Alzheimer's disease rat model [14]. Administration of sodium selenite together with melatonin 30 min before medial cerebral artery occlusion (MCAO) and for 3 days postreperfusion decreases oxidative stress [15]. A mechanism for the antioxidant effect of selenium is the inhibition of inducible nitric oxide synthases (iNOS) and COX-2 expression through the inactivation of p38 MAPK and NF-*κ*B [16]. Another mechanism is the incorporation of selenium into selenoproteins such as glutathione peroxidase and thioredoxin reductase, thus making the removal of peroxides more efficient [17].

Our group has reported opposite effects of zinc administration in the 10 min common carotid artery occlusion (CCAO) rat model, where CCAO causes cell death by apoptosis and necrosis without producing an ischemic core [11, 18]. The subacute administration of zinc (2.5 mg/kg) before CCAO exerts a neuroprotective effect by increasing the expression of CCL2/CCR2, FGF2, and IGF-1 in the temporoparietal rat cortex [5]. In contrast, chronic zinc administration at a low dose (0.5 mg/kg body weight) before CCAO decreases CCL2/CCR2, CCL3/CCR1, CCL4/CCR8, and CXCL13/CXCR5 and increases CXCL12/CXCR4, but does not prevent cell death in the late phase [19]. The antioxidant effect of selenium has been used to exert neuroprotection alone [12] or in combination with other antioxidants such as melatonin [15], *Ginkgo biloba* [13], and alpha-tocopherol [20]. Therefore, we propose that the combination of the prophylactic effect of zinc with the therapeutic effect of selenium can maintain the neuroprotection on neuroinflammation and neurodegeneration induced by transient CCAO.

To test that hypothesis, we administered zinc (0.2 mg/kg body weight; ip) for 14 days before CCAO, followed by sodium selenite (6 *μ*g/kg body weight; ip) administration for 7 days after CCAO. We determined nitrosative-oxidative stress (nitrites, lipid peroxidation, NOSs, and antioxidant enzymes), markers of neuroinflammation (chemokines and their receptors) and cell death, over time after CCAO. We also measured neuronal plasticity using the Morris-Water maze test. Because the temporoparietal cortex resulted to be more affected than the hippocampus, we only reported the findings in the temporoparietal cortex. Our results demonstrate that the combined treatment of zinc with selenium extends the effective neuroprotection against CCAO-induced hypoxia-ischemia.

## 2. Materials and Methods

### 2.1. Experimental Animals

Male Wistar rats (body weight 190 to 240 g) were obtained from the vivarium of CINVESTAV and maintained in suitable rooms with controlled conditions of temperature (22 ± 3°C) and light-dark cycles (12 h–12 h; light onset at 07 : 00). Five rats per cage (acrylic; 34 cm × 44 cm × 20 cm) were housed. Food (Laboratory Autoclavable Rodent Diet 5010, 130 ppm of zinc, and 0.47 ppm of selenium) and drinking water were provided ad libitum. All procedures were by the current Mexican legislation, NOM-062-ZOO-1999 (SAGARPA), which in turn is based on the Guide for the Care and Use of Laboratory Animals, NRC. The Institutional Animal Care and Use Committee approved the experimental procedures with the protocol number 09-102. All efforts were made to minimize animal suffering.

### 2.2. Zinc and Selenium Administration

Zinc was administered as ZnCl_2_ (0.2 mg/kg of body weight in water for injection, ip; Sigma-Aldrich; Saint Louis, MO, USA) every day for 14 days (chronic zinc administration). Sodium selenite (6 *μ*g/kg of body weight in the water for injection, ip Sigma-Aldrich; Saint Louis, MO, USA) administration started 24 h after the last dose of Zn and continued every day for 7 days. Rats were grouped as follows: (1) control, healthy rats without treatment and surgery, (2) zinc, chronic zinc administration, (3) Zn + Se, chronic zinc administration followed by a single selenium administration on day 15, (4) CCAO, common carotid artery occlusion for 10 min, (5) Zn + CCAO, chronic zinc administration before CCAO, and (6) Zn + CCAO + Se, chronic zinc administration before CCAO followed by selenium administration for 7 days. The rats of groups (1), (2), and (3) were euthanized on day 15 to dissect out their brains. The brains of groups (4), (5), and (6) were obtained at different times (3, 6, 24, and 168 h) after reperfusion. All the variables studied were measured in the temporoparietal cortex, and all rat groups were age-matched.

### 2.3. Common Carotid Artery Occlusion (CCAO)

The asepsis procedures were performed in the surgical instruments and surgical area. The animals were anesthetized with a mixture of ketamine (70 mg/kg) and xylazine (6 mg/kg) at a dose of 200 *μ*L/100 g of body weight, ip. A 0.5 cm-long midline skin incision was made in the neck area, and the left common carotid artery was carefully dissected. Then, the artery was occluded for 10 min with a clamp (Bulldog Clamps, INS6000119; Kent Scientific Corporation; Torrington, CT, USA). Upon completion of the occlusion, the reperfusion of the artery was visually verified, and the incision was sutured with a 3-0 silk thread (Atramat; Ciudad de Mexico, Mexico). The animals were kept in an individual cage under a 100-Watt, yellow light source until their complete recovery. The animals were euthanized and beheaded in the corresponding time postreperfusion using ketamine (70 mg/kg) and xylazine (6 mg/kg) at a dose of 200 *μ*L/100 g of body weight. The ipsilateral temporoparietal cortex from the different groups was obtained for the biochemical, cellular, and molecular assays.

### 2.4. Nitrites

The temporoparietal cortex (*n* = 5 rats in each group) was mechanically homogenized in phosphate-buffered saline solution (PBS), pH 7.4, and centrifuged at 12,500 rpm for 30 min at 4°C by using a Z 216 MK microcentrifuge (HERMLE Labortechnik; Wehingen, Germany). The production of NO was assessed through the accumulation of nitrites (NO_2_^−^) in the supernatants as described elsewhere [21]. Briefly, the nitrite concentration in 100 *μ*L of the supernatant was measured by using a colorimetric reaction generated by the addition of 100 *μ*L of Griess reagent, composed of equal volumes of 0.1% N-(1-naphthyl)ethylenediamine dihydrochloride and 1.32% sulfanilamide in 60% acetic acid. The absorbance of the samples was determined at 540 nm with a SmartSpec 3000 spectrophotometer (Bio-Rad; Hercules, CA, USA) and interpolated by using a standard curve of NaNO_2_ (1 to 10 *μ*M) to calculate the nitrite concentration.

### 2.5. Lipid Peroxidation

Malondialdehyde (MDA) and 4-hydroxyalkenals (4-HAD) were measured in the same supernatant where the nitrites were measured (*n* = 5 rats in each group), following the procedure described elsewhere [22]. The colorimetric reaction was made using 200 *μ*L of the supernatant after the subsequent addition of 650 *μ*L of 10.3 mM N-methyl-2phenyl-indole (Sigma-Aldrich; Saint Louis, MO, USA) diluted in a mixture of acetonitrile : methanol (3 : 1) and 150 *μ*L of methanesulfonic acid (Sigma-Aldrich; Saint Louis, MO, USA). The reaction mixture was vortexed and incubated at 45°C for 1 h and afterward centrifuged at 3000 rpm for 10 min. The absorbance in the supernatant was read at 586 nm with a SmartSpec 3000 spectrophotometer (Bio-Rad; Hercules, CA, USA). The absorbance values were compared to a standard curve in the concentration range of 0.5 to 5 *μ*M of 1,1,3,3-tetramethoxypropane (10 mM stock) to calculate the content of malondialdehyde + 4-hydroxyalkenal (MDA + 4-HAD) in the samples.

### 2.6. Glutathione Peroxidase Activity

GPx activity was measured using the Glutathione Peroxidase Assay Kit (ab102530; Abcam; Cambridge, UK) following the manufacturer's instructions. A sample of 50 mg of the temporoparietal cortex was washed with cold PBS and homogenized in 200 *μ*L cold assay buffer with a mechanic homogenizer on ice for 15 passes and centrifuged at 10000*g* for 15 min at 4°C. The supernatants were collected into a fresh corresponding tube and samples of 50 *μ*L were distributed in the respective wells of ELISA microplates (Costar, Corning Incorporated; NY, USA). Then, 40 *μ*L of the fresh reaction mix was added to the well, mixed, and incubated at room temperature for 15 min. Then, 10 *μ*L of cumene hydroperoxide solution was added to each well, mixed, and read at 340 nm on a plate reader (Bio-Rad; Hercules, California, USA). Then, the plates were incubated in the dark at 25°C for 5 min and read again at 340 nm. The activity of GSH-Px in nmol/mL was calculated according to the manufacturer's instructions.

### 2.7. Superoxide Dismutase Activity

SOD activity was measured using the Superoxide Dismutase Activity Colorimetric Assay kit (ab65354, Abcam; Cambridge, UK) following the manufacturer's instructions. A temporoparietal cortex sample of 50 mg from each rat group was washed in cold PBS and homogenized with a mechanic homogenizer on ice for 15 passes in cold 0.1 M Tris/HCl, pH 7.4 (containing 0.5% Triton X-100, 5 mM *β*-ME, and 0.1 mg/mL PMSF). Then, the homogenates were centrifuged for 5 min at 4°C at 14000*g*. The supernatants were collected into a fresh corresponding tube, and samples of 20 *μ*L were distributed in the respective wells of ELISA microplates (Costar, Corning Incorporated; NY, USA). Then, 200 *μ*L of WST working solution was added into each well followed by the addition of 20 *μ*L of Enzyme Working Solution. The plates were mixed and incubated for 20 min at 37°C before reading using a microplate reader (Bio-Rad; Hercules, California, USA) at 450 nm. The OD of each sample was normalized with untreated control (group 1).

### 2.8. Enzyme-Linked Immunosorbent Assay (ELISA)

CCL2/CCR2, CXCL12/CXCR4, and CXCL13/CXCR5 levels were measured by ELISA in homogenates of the temporoparietal cortex (*n* = 5 for each group). Protein content was determined using the Sedmak and Grossberg method [23]. Aliquots containing 5 *μ*g of total protein were placed into wells of ELISA plates. Subsequently, 100 *μ*L of 0.1 M carbonate buffer was added into each well and the plates were incubated at 4°C for 18 h. To block nonspecific binding sites, 200 *μ*L of 0.5% bovine serum albumin (IgG free) was added into each well at room temperature. After 30 min incubation, the wells were washed thrice with PBS-Tween 20 (0.1%) solution. The primary antibodies were rabbit monoclonal antibodies to CCL2 (Bio-Rad/AbD Serotec Cat. number AAR31, RRID:AB_2071792, 1 : 500 dilution), and the following antibodies were obtained from Abcam (Abcam; Cambridge, UK), CCR2 (Abcam Cat. number ab21667, RRID:AB_446468, 1 : 500 dilution), CXCL12 (Abcam Cat. number ab25118, RRID:AB_448630, 1 : 500 dilution), CXCL13 (Abcam Cat. number ab112521, RRID:AB_10863283, 1 : 500 dilution), CXCR4 (Abcam Cat. number ab2074, RRID:AB_302814, 1 : 500 dilution), and CXCR5 (Abcam Cat. number ab10405, RRID:AB_2089665, 1 : 500 dilution). The primary antibodies were added to each well and incubated for 2 h at room temperature. After three washes with PBS-Tween 20 (0.1%), a horseradish-peroxidase-conjugated goat anti-rabbit or mouse IgG (1 : 1000 dilution; Dako North America Inc; Carpinteria, CA, USA) was added into the wells and incubated for 2 h at room temperature. The antibody-antigen complex was revealed by addition of 100 *μ*L of 2,2′-azino-bis(3-ethylbenzthiazoline-6-sulfonic acid) (ABTS) containing 0.3% H_2_O_2_ into each well. After 15 min, optical density (OD) was determined using a Benchmark multiple reader at 415 nm (Bio-Rad; Hercules, CA, USA). All samples were processed under the same experimental conditions and time.

### 2.9. Retrotranscription

Total RNA was isolated from 100 mg of temporoparietal cortex using 1 mL of TRIzol (Invitrogen Corporation; Carlsbad, CA, USA) and then RNA-treated with RNase-free DNase I and quantified using a NanoDrop Spectrophotometer (Thermo Scientific NanoDrop Technologies; Wilmington, DE, USA). cDNA was obtained from 5 *μ*g of total RNA using 1 *μ*L of SuperScript III reverse transcriptase kit (Catalog 18080093, Invitrogen; Carlsbad, CA, USA),1 *μ*L of Oligo dT 50 *μ*M, 1 *μ*L of dNTP mix 10 mM, and water grade molecular biology to 13 *μ*L. Retrotranscription conditions were denaturation at 70°C for 10 min, hybridization at 42°C for 5 min, synthesis of cDNA at 55°C for 50 min and then 70°C for 15 min, and removal of RNA at 37°C for 20 min. Finally, 1 *μ*L of RNase H (Invitrogen; Carlsbad, CA, USA) was added and samples were incubated at 37°C for 20 min.

### 2.10. qPCR

Fresh cDNA was used to amplify each gene using TaqMan probes (Table 1) obtained from Thermo Fisher (Thermo Fisher Scientific; Waltham, MA, USA). The amplification reactions contained 0.25 *μ*L of the respective TaqMan probe, 2.5 *μ*L of Master Mix (TaqMan Universal Master Mix; Life Technologies; Carlsbad, CA, USA), and 2.25 *μ*L of cDNA in a final volume of 5 *μ*L. The conditions for qPCR were 10 min for denaturation at 95°C, followed by 45 cycles of amplification of 15 s at 95°C and 1 min at 60°C. Rat *β*-actin was used as internal control and for normalization. The amplification assays were made using a 7900HT Fast Real-Time PCR System (Applied Biosystems; Foster City, CA, USA). The 2^−ΔΔCt^ analyses were used to calculate the relative transcript levels expressed as fold change for gene expression.

### 2.11. Spatial Reference Learning and Memory

The Morris water maze was used to measure the spatial reference memory. The measurements were conducted in a round tank, 150 cm in diameter and 80 cm deep, filled with water, and divided into four imaginary quadrants. Water was maintained at a temperature of 23 ± 2°C. Several distal visual cues were placed on both walls of the Morris water maze and the room in which it had been installed. This evaluation consisted of five test days of four consecutive trials per day. During the trial, each animal was placed in the tank facing the wall and allowed to swim freely to an escape platform (40 cm in height and 15 cm in diameter), which was submerged by 2 cm under the water surface and conserved to the center of the southeast (SE) quadrant of the tank. If the animals did not find the platform during a period of 60 s in the first trial of each test day, they were gently guided to it, allowed to remain on the platform for 30 s, and then removed from the tank. This procedure was used to ensure that the animals retained the visual-spatial information of the maze online during the execution of the swimming task [24]. Long-term memory was evaluated in the absence of the platform on day 7 after learning. The latency to reach the platform and the number of times that rats pass by the platform location were measured.

### 2.12. Histopathological Study

The animals were anesthetized with ketamine (70 mg/kg) and xylazine (6 mg/kg) at a dose of 200 *μ*L/100 g of body weight, ip, and intracardially perfused with 200 mL of PBS followed by 100 mL of 4% paraformaldehyde. The brains were obtained and kept in 4% paraformaldehyde at 4°C for 24 h. The brains were embedded in paraffin using a Histokinette (Leica Microsystems; Wetzlar, Germany). The steps of the tissue processing were consecutive dehydration in different ethanol concentrations (80% for 1 h, 96% for 3 h, and 100% for 3), clearance in pure xylol for 2 h, and inclusion in paraffin at 56°C for 2 h. The tissues were placed in blocks using metallic cassettes. The histological sections of 3 *μ*m were made in a rotary microtome-type Minot (Leica RM2135; Wetzlar, Germany) and placed on slides recovered with poly-L-lysine and finally fixed with heat at 60°C for 30 min. The slices were deparaffinized in an oven at 60°C and placed in xylene 2 times for 15 min. The hydration of slices was made by two consecutive passages in decreasing concentration of ethanol (100%, 96%, and 80%) and finally in tap water for 5 min. Hematoxylin staining was performed for 5 min or less until sections look blue. After washing with tap water, the differentiation was carried out with 1% acid alcohol (1% HCl in 70% alcohol) for 1 dip. The slices were washed in running tap water and dipped again in an alkaline solution (i.e., a saturated solution of lithium carbonate) followed by a tap water washing. The staining in 1% eosin Y was done for 10 minutes. The slices were washed again with tap water for 1 min. The dehydration of slices was made by ten consecutive dippings in increasing concentration of alcohols (80%, 96%, and 100%) and clearing with xylene. Finally, the slices were mounted on glass slides using Entellan (Merck KGaA; Darmstadt, Germany) and protected with coverslips. The slides were then examined with a light microscope equipped with 10x objective (Leica Microsystems; Wetzlar, Germany). The count of pyknotic cells in 20 fields of 4 brains in layer 5 of the temporoparietal cortex was made using ImageJ software (RRID:SCR_003070, National Institutes of Health).

### 2.13. Experimental Design and Statistical Analysis

All values were expressed as mean ± SEM from 5 independent experiments including the controls. The values of each variable studied were normalized concerning group 1 (untreated control), except the qPCR values that were expressed as a fold change (2^−ΔΔCt^). One-way ANOVA and Dunnett's post hoc test were used to compare all groups with the untreated control group. Student's *t*-test was used to compare the treated groups concerning the CCAO group. All statistical analyses were performed using the GraphPad Prism 6 software. The results of learning memory were analyzed with Kruskal-Wallis one-way analysis of variance for comparison of multiple groups, and the Mann-Whitney *U* test was used for the statistical analysis of two groups. *P* values <0.05 were considered statistically significant. All statistical analyses were performed using data analysis software (GraphPad Prism, RRID:SCR_0158070).

## 3. Results

**Figure 1:**
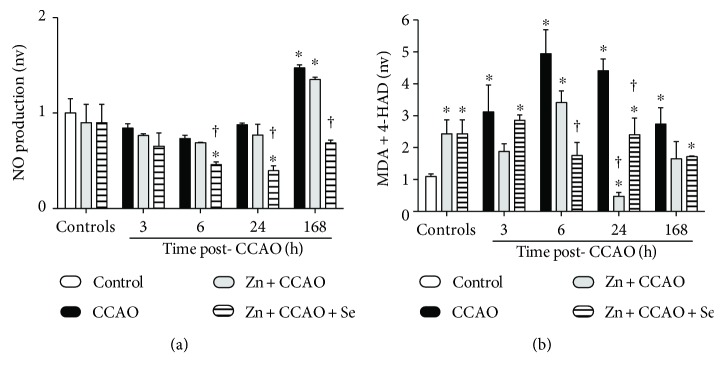
The combined treatment with zinc and selenium decreases the CCAO-induced nitrosative-oxidative stress in the temporoparietal cortex. (a) Nitrosative stress and (b) lipid peroxidation. CCAO: common carotid artery occlusion for 10 min; Zn + CCAO: chronic zinc administration before CCAO; Zn + CCAO + Se: chronic zinc administration before CCAO followed by selenium administration. The values were normalized against the control untreated group; nv: normalized values. Each value represents mean ± SEM of 5 independent experiments made in triplicate. ^∗^*P* < 0.05, one-way ANOVA with post hoc Dunnett's test when compared to the control untreated group. ^†^*P* < 0.05, Student's *t*-test when compared with the respective CCAO group.

CCAO increased NO production at 168 h (43 ± 2%, *P* = 0.0001) postreperfusion in the temporoparietal cortex when compared with the untreated control (Figure 1(a)). The Zn + CCAO group did not prevent the CCAO-induced increase at 168 h in nitrite levels (35 ± 2%, *P* = 0.0001) when compared with the untreated control (Figure 1(a)). In contrast, the combined treatment with zinc and selenium (Zn + CCAO + Se) completely normalized the nitrite levels when compared with the CCAO group (Figure 1(a)), suggesting a reduction of nitrosative stress. In addition, this combined treatment reduced nitrite levels at 6 h (27 ± 4%, *P* = 0.0036), 24 h (54 ± 6%, *P* = 0.0009), and 168 h (53 ± 2% *P* = 0.0004) after CCAO, when compared to the respective CCAO group (Figure 1(a)).

CCAO also increased lipid peroxidation in the temporoparietal cortex from 3 h (211 ± 84%, *P* = 0.0405), with a maximum peak at 6 h (340 ± 37%, *P* = 0.0010) postreperfusion (Figure 1(b)). The zinc group caused a preconditioning effect, generating an increase in MDA + 4-HAD levels (123 ± 30%, *P* = 0.0130) when compared with the untreated control (Figure 1(b)). The Zn + CCAO group decreased the CCAO-induced increase in lipid peroxidation (89 ± 2%, *P* = 0.0492) only at 24 h, whereas the combined treatment Zn + CCAO + Se decreased lipid peroxidation levels as early as 6 h after CCAO until the end of the study as compared with the respective CCAO group (Figure 1(b)). The reduction in MDA + 4-HAD levels in the Zn + CCAO + Se group at 6 h was 64 ± 8% (*P* = 0.0260), at 24 h was 44 ± 12% (*P* = 0.0362), and at 168 h was 37 ± 1% (*P* = 0.0285).

**Figure 2:**
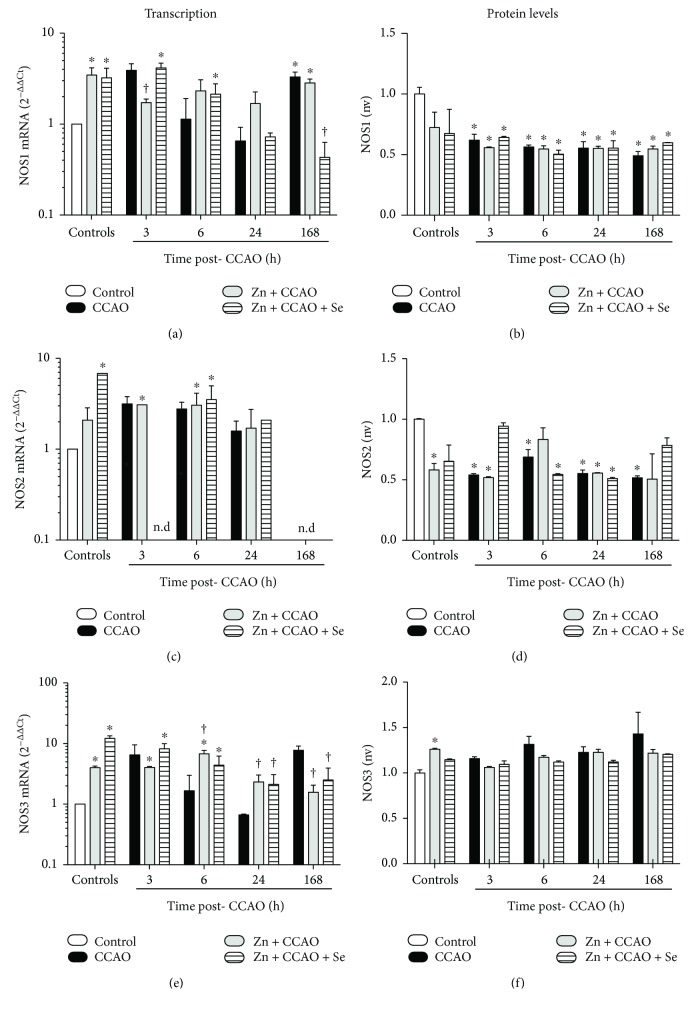
Effect of the combined treatment with zinc and selenium on nitric oxide synthase transcription and translation in the temporoparietal cortex of ischemic rats. (a), (c), and (e) mRNA levels; (b), (d), and (f) protein levels. CCAO: common carotid artery occlusion for 10 min; Zn + CCAO: chronic zinc administration before CCAO: Zn + CCAO + Se: chronic zinc administration before CCAO followed by selenium administration. The values were normalized against the control group. nv: normalized values. Each value represents mean ± SEM of 5 independent experiments made in triplicate. ^∗^*P* < 0.05, one-way ANOVA with post hoc Dunnett's test when compared to the control untreated group; ^†^*P* < 0.05, Student's *t*-test when compared with the respective CCAO group.

We evaluated the transcription and expression of NOSs to determine their participation in the NO production in the temporoparietal cortex (Figure 2). CCAO increased mRNA levels for *Nos1* (Figure 2(a)) and *Nos3* (Figure 2(e)), but not for *Nos2* (Figure 2(c)). The increase was statistically significant at 3 h (3.89 ± 0.72, *P* = 0.0157) for *Nos1* and 6.43 ± 3.05, *P* = 0.0358, for *Nos3* at 168 h (3.3 ± 0.4, *P* = 0.0499 for *Nos1* and 7.7 ± 1.1 for *Nos3*, *P* = 0.0147) after CCAO (Figures 2(a) and 2(e), resp.). An upregulation of *Nos1* (3.4 ± 0.69 fold change, *P* = 0.0497) and *Nos3* (12.1 ± 1.25 fold change, *P* = 0.0289) mRNAs was induced by the Zn + Se group; the latter administration also upregulated Nos2 mRNA (6.82 ± 0.1 fold change, *P* = 0.0001) (Figure 2(c)). Zn + CCAO only prevented the CCAO-induced increase in *Nos1* mRNA at 3 h after CCAO and in *Nos3* mRNA at 168 after CCAO (Figure 2(a) and Figure 2(e)). The combined treatment Zn + CCAO + Se prevented the CCAO-induced increase in *Nos1* and Nos3 mRNAs only at 168 h after CCAO (Figures 2(a) and 2(e)). It is interesting to note that *Nos2* mRNA was not detected in the Zn + CCAO + Se group at 3 h and 168 h after CCAO (Figure 2(c)). A similar effect occurred in the CCAO group and Zn + CCAO group at 168 h after CCAO (Figure 2(c)).

Concerning NOSs protein expression, the CCAO do not modify NOS3 (Figure 2(f)). NOS1 (Figure 2(b)) and NOS2 (Figure 2(d)) in the temporoparietal cortex decreased their expression over time in all groups when compared with the untreated controls, and there was no statistical difference among the groups. The average of decrease was 43% (*P* < 0.004) for NOS1 and 44% (*P* < 0.005) for NOS2.

**Figure 3:**
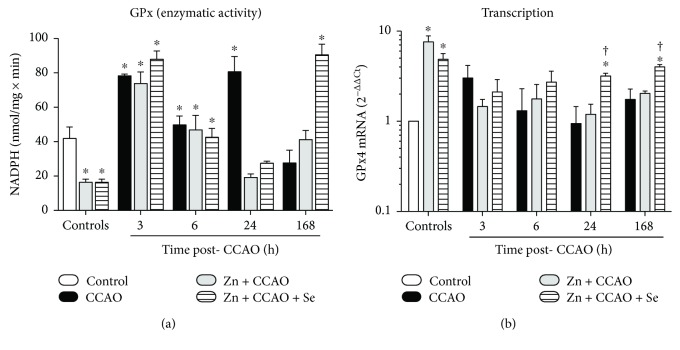
The combined treatment with zinc and selenium increased the enzymatic activity and transcription of glutathione peroxidase in the late phase of hypoxia-ischemia in the rat. (a) Enzymatic activity of GPx and (b) mRNA levels of GPx4. CCAO: common carotid artery occlusion for 10 min; Zn + CCAO: chronic zinc administration before CCAO; Zn + CCAO + Se: chronic zinc administration before CCAO followed by selenium administration. The values were normalized against the control group. Each value represents mean ± SEM of 5 independent experiments made in triplicate. ^∗^*P* < 0.05, one-way ANOVA with post hoc Dunnett's test when compared to the control untreated group; ^†^*P* < 0.05, Student's *t*-test when compared with the respective CCAO group.

CCAO increases GPx activity in the temporoparietal cortex at 3 h (86 ± 2%, *P* = 0.0132) and at 24 h postreperfusion (92 ± 11%, *P* = 0.0100) when compared with the untreated controls (Figure 3(a)). The Zn + Se group significantly decreased at the same levels (60 ± 4%, *P* = 0.0311) for GPx activity (Figure 3(a)). A significant decrease in GPx activity was observed at 24 h postreperfusion (Figure 3(a)) in the Zn + CCAO (76 ± 3%, *P* = 0.0027) and Zn + CCAO + Se groups (65 ± 2%, *P* = 0.0043). At 168 h postreperfusion, only the Zn + CCAO + Se group increased by 227 ± 22% for GPx (*P* = 0.0028) activity when compared with the respective CCAO group (Figure 3(a)), showing an antioxidant effect in the late phase.

In the absence of CCAO, *Gpx4* mRNA was upregulated with zinc administration (7.6 ± 1.25, *P* = 0.0008) and Zn + Se administration (4.8 ± 0.7, *P* = 0.0190) when compared with the untreated controls (Figure 3(b)). CCAO did not affect the basal levels of *Gpx4* mRNA (Figure 3(b)). The combined treatment Zn + CCAO + Se increased *Gpx4* mRNA at 24 h (3.35 ± 0.25, *P* = 0.0459) and at 168 h (2.3 ± 0.13, *P* = 0.0485) postreperfusion (Figure 3(c)) as compared with the CCAO group (Figure 3(b)).

**Figure 4:**
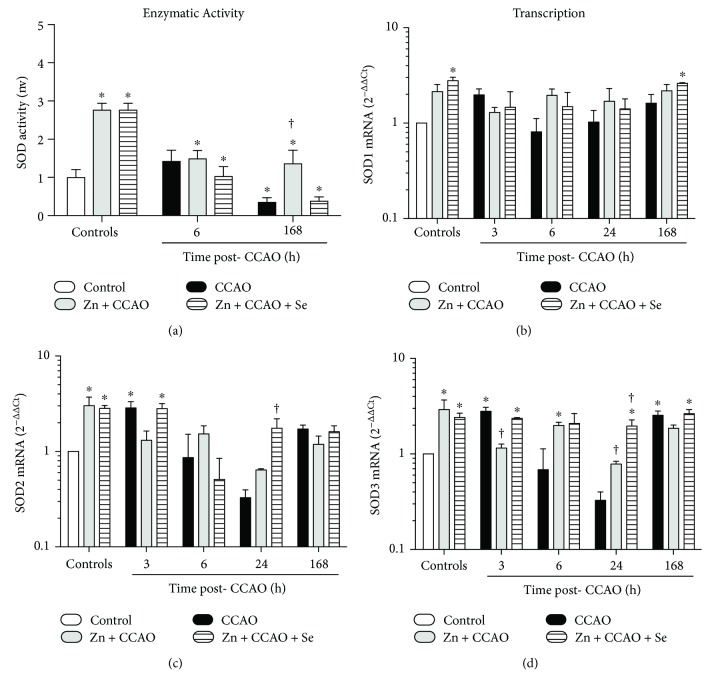
Differential effect of the combined treatment with zinc and selenium on the enzymatic activity and transcription of superoxide dismutase in the rat hypoxia-ischemia model. (a) Enzymatic activity of SOD, (b) mRNA levels of Sod1, (c) mRNA levels of Sod2, and (d) mRNA levels of Sod3. CCAO: common carotid artery occlusion for 10 min; Zn + CCAO: chronic zinc administration before CCAO; Zn + CCAO + Se: chronic zinc administration before CCAO followed by selenium administration. The values were normalized against the control group. Each value represents mean ± SEM of 5 independent experiments made in triplicate. ^∗^*P* < 0.05, one-way ANOVA with post hoc Dunnett's test when compared to the control untreated group; ^†^*P* < 0.05, Student's *t*-test when compared with the CCAO group.

SOD activity in the temporoparietal cortex was increased at 258 ± 12% (*P* = 0.0021) by the zinc or Zn + Se group when compared with untreated controls (Figure 4(a)). CCAO decreases SOD activity (65 ± 11%, *P* = 0.0486) at 168 h when compared with the untreated controls (Figure 4(a)). At this time, only Zn + CCAO increased SOD activity (78 ± 20%, *P* = 0.0457) when compared with the respective CCAO group (Figure 4(a)).

The zinc or Zn + Se group is differentially regulated in the *Sod* isoforms. The zinc group did not affect *Sod1* transcript levels (Figure 4(b)), whereas it upregulated *Sod2* (3.0 ± 0.69, *P* = 0.0453) and Sod3 (2.9 ± 0.76, *P* = 0.0280) transcripts when compared with the untreated controls (Figures 4(c) and 4(d)). The increases in mRNA levels were 2.78 ± 0.23-fold (*P* = 0.0030) for *Sod1*, 2.8 ± 0.2-fold (*P* = 0.0395) for *Sod2*, and 2.4 ± 0.3-fold (*P* = 0.0383) for *Sod3*. CCAO did not affect *Sod1* transcripts. Whereas CCAO upregulated *Sod2* (2.9 ± 0.46, *P* = 0.0422) at 3 h (Figure 4(c)) and *Sod3* at 3 h (2.8 ± 0.28, *P* = 0.0038) and 168 h (2.5 ± 0.39, *P* = 0.0065), CCAO did not modify when compared with the untreated controls (Figure 4(d)). None of the treatments modified *Sod1* transcripts in the times studied when compared with the respective CCAO group (Figure 4(b)). Zn + CCAO + Se only upregulated *Sod2* (5.3 ± 1.3, *P* = 0.0051) and *Sod3* (6.13 ± 0.9, *P* = 0.0290) at 24 h postreperfusion when compared to the respective CCAO group (Figures 4(c) and 4(d)).

**Figure 5:**
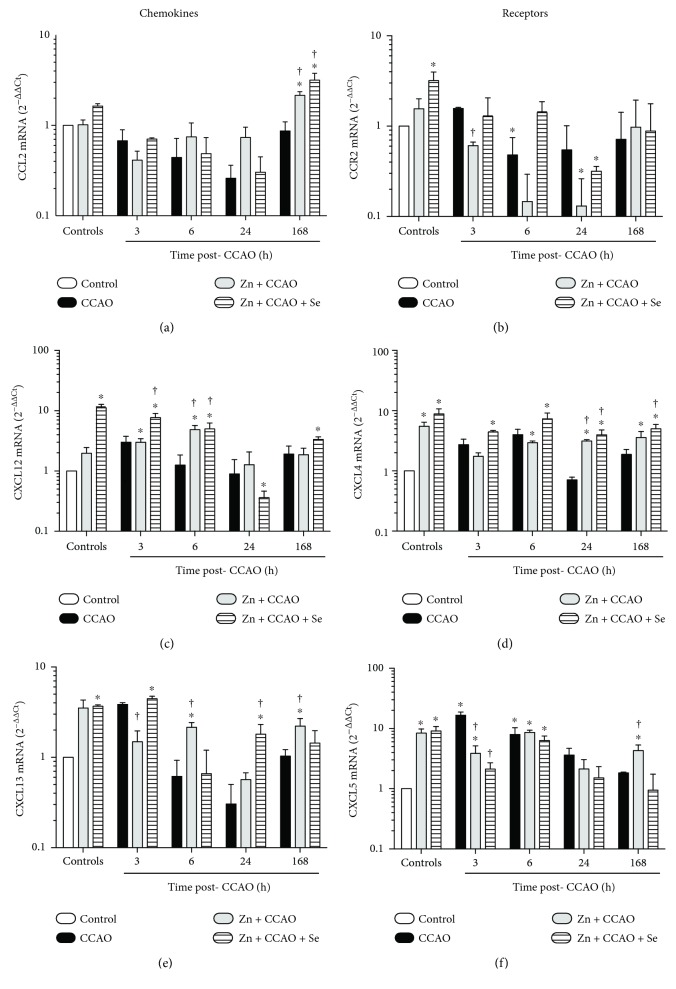
Differential effect of the combined treatment with zinc and selenium on chemokine and receptor transcription in the rat hypoxia-ischemia model. (a), (c), and (e) Chemokines levels; (b), (d), (f) receptors of the respective chemokines. CCAO: common carotid artery occlusion for 10 min; Zn + CCAO: chronic zinc administration before CCAO; Zn + CCAO + Se: chronic zinc administration before CCAO followed by selenium administration. The values were normalized against the control group. Each value represents mean ± SEM of 5 independent experiments made in triplicate. ^∗^*P* < 0.05, one-way ANOVA with post hoc Dunnett's test when compared to the control untreated group; ^†^*P* < 0.05, Student's *t*-test when compared with the respective CCAO group.

**Figure 6:**
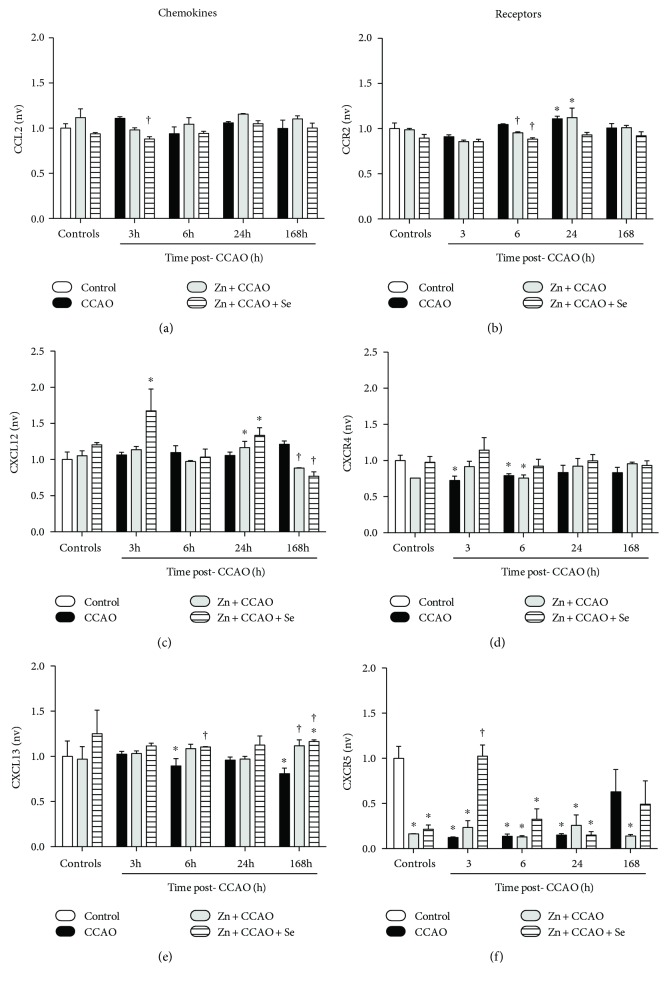
The administration of the combined treatment with zinc and selenium modifies the protein levels of chemokines and receptors in the rat hypoxia-ischemia model. (a), (c), and (e) Chemokine levels, and (b), (d), (f) receptors of the respective chemokines. CCAO: common carotid artery occlusion for 10 min; Zn + CCAO: chronic zinc administration before CCAO. Zn + CCAO + Se: chronic zinc administration before CCAO followed by selenium administration. The values were normalized against the control group. nv: normalized values. Each value represents mean ± SEM of 5 independent experiments made in triplicate. ^∗^*P* < 0.05, one-way ANOVA with post hoc Dunnett's test when compared with the untreated control group; ^†^*P* < 0.05, Student's *t*-test when compared with the respective CCAO group.

Chemokine transcription levels in the temporoparietal cortex are shown in Figure 5. CCAO did not affect transcription of *Ccl2* and its receptor *Ccr2* in the time studied (Figures 5(a) and 5(b)). As compared with the control group, CCAO upregulated the following chemokines and their receptors: *Cxcl12* at 3 h (3 ± 0.7, *P* = 0.0426; Figure 5(c)), its receptor *Cxcr4* at 3 h (2.7 ± 0.61, *P* = 0.0475; Figure 5(d)) and 6 h post-CCAO (3.9 ± 0.9, *P* = 0.0354; Figure 5(d)), and *Cxcl13* at 3 h (3.84 ± 0.0204, *P* = 0.01; Figure 5(e)) and its receptor *Cxcr5* at 3 h (16.6 ± 2.2, *P* = 0.0005; Figure 5(f)) and at 6 h (8.0 ± 2.3, *P* = 0.0316; Figure 5(f)).

Zinc caused an upregulation only of *Cxcr4* (5.5 ± 0.9, *P* = 0.0354; Figure 5(d)), *Cxcl13* (3.5 ± 0.8, *P* = 0.0218; Figure 5(e)), and *Cxcr5* (8.3 ± 1.4, *P* = 0.0261; Figure 5(f)), whereas Zn + Se upregulated *Ccr2* (3.19 ± 0.77, *P =* 0.0164, Figure 5(b)), *Cxcl12* (11.2 ± 1.56, *P* = 0.0001; Figure 5(c)), *Cxcr4* (8.8 ± 1.8, *P* = 0.0006, Figure 5(d)), *Cxcl13* (3.6 ± 0.13, *P* = 0.0268; Figure 5(e)), and *Cxcr5* (9.1 ± 1,6, *P* = 0.0085; Figure 5(f)).

As compared with CCAO effect, Zn + CCAO upregulated the following chemokines and their receptors: *Ccl2* at 168 h (2.4 ± 0.2, *P* = 0.0307; Figure 5(a)), *Cxcl12* at 6 h (3.8 ± 0.69, *P* = 0.0376; Figure 5(c)), and *Cxcr4* at 24 h (4.4 ± 0.2, *P* = 0.0001; Figure 5(d)). Upregulation and downregulation were observed in *Cxcl13* at 3 h (0.38 ± 0.12, *P* = 0.0321) and at 6 h (3.49 ± 0.44, *P* = 0.0428; Figure 5(e)) and in *Cxcr5* at 3 h (0.23 ± 0.07, *P* = 0.0075) and at 168 h (2.3 ± 0.55, *P* = 0.0473; Figure 5(f)).

As compared with the CCAO group, Zn + CCAO + Se caused an upregulation of *Ccl2* at 168 h (3.7 ± 0.69, *P* = 0.0226; Figure 5(a)) and *Cxcl12* at 3 h (2.5 ± 0.43, *P* = 0.0367) and 6 h (4 ± 1, *P* = 0.0314, Figure 5(c)). *Cxcr4* was upregulated since 24 h (5.5 ± 1.1, *P* = 0.0162) to 168 h (2.65 ± 0.49, *P* = 0.0413; Figure 5(d)) post-CCAO. *Cxcl13* was upregulated at 24 h (5.9 ± 1.6, *P* = 0.0357; Figure 5(e)), and *Cxcr5* was downregulated at 3 h (0.12 ± 0.03, *P* = 0.0031; Figure 5(f)).

Protein levels of CCR2 were increased by CCAO at 24 h (17 ± 6%, *P* = 0.0457; Figure 6(b)) in the temporoparietal cortex. CXCL12 levels were increased by Zn + CCAO at 24 h (24 ± 4%, *P* = 0.0114; Figure 6(c)) or Zn + CCAO + Se at 24 h (32 ± 6%, *P* = 0.0026; Figure 6(c)), whereas CXCL13 levels were increased by Zn + CCAO + Se (16 ± 1%, *P* = 0.0425; Figure 6(e)) at 168 h post-CCAO.

**Figure 7:**
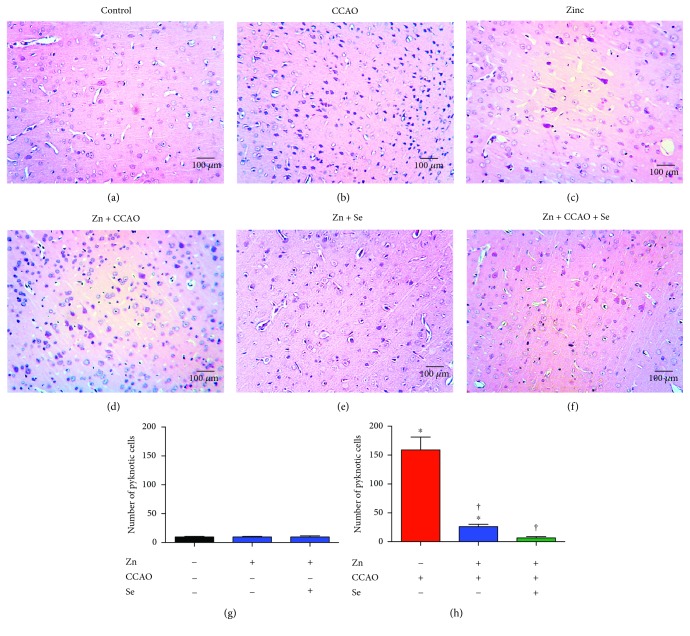
The administration of the combined treatment with zinc and selenium decreases the number of pyknotic cells in the temporoparietal cortex of hypoxia-ischemic rats. Representative micrographs with hematoxylin-eosin staining. The headings indicate the different experimental conditions. (g) Counting of pyknotic cells in the control groups treated with zinc or zinc + Se. (h) Counting of pyknotic cells in the groups with CCAO and treated with zinc (zinc) or the combined treatment with zinc (zinc + CCAO) and selenium (zinc + CCAO + Se). Each value represents mean ± SEM of 5 independent experiments made in triplicate. ^∗^*P* < 0.05, one-way ANOVA with post hoc Dunnett's test when compared with the untreated control group; ^†^*P* < 0.05, Student's *t*-test when compared with the CCAO group.

The histopathology studies showed the presence of pyknotic cells at 168 h after CCAO (Figure 7(b)) as compared with the control group (Figure 7(a)). Zinc (Figure 7(c)) or Zn + Se (Figure 7(e)) did not modify the histological morphology as compared with the untreated control. On the contrary, CCAO caused a significant increase in the number of pyknotic cells (indicative of apoptosis) by 1460 ± 188% (*P* = 0.0001) at 168 h postreperfusion as compared with the untreated control (Figures 7(b) and 7(h)). Zn + CCAO reduced the number of pyknotic cells by 80% ± 3% (*P* = 0.001) at 168 h, as compared with the CCAO group (Figures 7(d) and 7(h)), but there was a statistical difference as compared with the untreated control group (Figure 7(g)). Zn + CCAO + Se significantly decreased the number of pyknotic cells by 90 ± 2% (*P* = 0.0001) as compared with CCAO (Figure 7(f) and 7(h)) and reached the basal values of the untreated control (Figure 7(g)).

**Figure 8:**
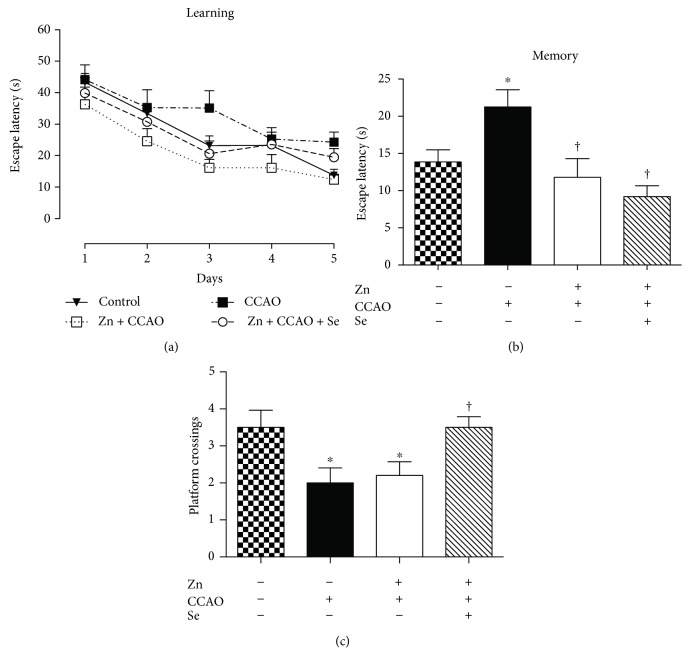
The administration of the combined treatment with zinc and selenium improves information consolidation in the hypoxia-ischemia model in rats. Control = untreated rats; Zn = zinc; CCAO = common carotid artery occlusion; Zn + CCAO = chronic zinc administration before CCAO; Zn + CCAO + Se = chronic zinc administration before CCAO followed by selenium (Se) administration. Each value represents mean ± SEM of 5 independent experiments made in triplicate. ^∗^*P* < 0.05, one-way Kruskal-Wallis analysis of variance with post hoc Dunnett's test when compared with the untreated control group; ^†^*P* < 0.05, Mann-Whitney *U* test when compared with the CCAO group.

The functional recovery from CCAO was assessed through the learning and memory test using the Morris water maze. There was not any statistical difference in information acquisition among the groups (Figure 8(a)). In memory evaluation, 7 days after the training, CCAO increased the escape latency by 53.9 ± 17% (*P* = 0.0238; Figure 8(b)) and decreased the crossing by the platform location by 37 ± 10% (*P* = 0.0272; Figure 8(c)) as compared with the controls. On the contrary, Zn + CCAO or Zn + CCAO + Se significantly decreased the latency time to remember the localization of the escape platform by 44 ± 11% (*P* = 0.0197) and 56 ± 7% (*P* = 0.0046), respectively, as compared with CCAO, suggesting improvement of consolidation of information. Only the Zn + CCAO + Se group showed a crossing by the platform location (65 ± 14% *P* = 0.0348) higher than CCAO, thus confirming that this treatment favors the neuronal functionality and exerts effective neuroprotection against hypoxia-ischemia.

## 4. Discussion

Our results show that the combined prophylactic of zinc and therapeutic of selenium administration had better effective protection against a transient hypoxic-ischemic event in the temporoparietal cortex, unlike other strategies we have tested such as the prophylactic administration of Se alone or combined with Zn (data not shown). This neuroprotection can be mainly explained by the increase in transcription and enzymatic activity of GPx and SOD, which prevented lipid peroxidation, and the significant decrease in neuronal cell death that is shown by the improvement of long-term memory.

Several studies have shown that chronic prophylactic administration of zinc shows a preconditioning effect [25–27]. This preconditioning effect can be explained by the induction of antioxidant enzymes, chemokines, and DNA methylases through zinc finger proteins [28–30]. Selenium has also been involved in the epigenetic regulation at least of antioxidant enzymes and DNA methylases [31]. Accordingly, our results show that the administration of those elements caused an upregulation of N*os3*, *Gpx4*, and *Sod* and the chemokines *Ccl2*, *Cxcl12/Cxcr4*, and *Cxcl13/Cxcr5*; the translation of NOS3; and the increase in the enzymatic activity of GPx and SOD. However, the protein levels of these chemokines and their receptors were not modified by zinc or selenium administration in the period studied. Therefore, the major contributor to the preconditioning effect in the transient hypoxia-ischemia model was the antioxidant effect and the preservation of NO bioavailability through NOS3 expression. As reported in a similar hypoxia-ischemia model, NOS3 is essential in the preservation and maintenance of microcirculation, inhibiting platelet aggregation, leukocyte adhesion, and migration and decreasing the inflammatory response [32]. The increased expression of NOS3 induced by zinc might be associated with the zinc finger protein ZFP580 [33]. Also, zinc stabilizes the dimerization of NOS3, which can prevent the production of superoxide anion and promote the increase in nitric oxide (NO) in the early phase of the hypoxia-ischemia process [34, 35]. Interestingly, selenium enhanced NOS3 expression similarly to only zinc administration at the early phase of CCAO, thus recovering the endothelial function as shown in an endothelial dysfunction model [36].

Our results showed that the prophylactic chronic zinc administration in the transient hypoxia-ischemia model increased the enzymatic activity of SOD and the levels of the transcripts of *sod1*, *sod2*, and *sod3.* These enzymes are known to play a major role in protecting from intracellular, extracellular, and mitochondrial oxidative stress, as reported in the cerebral cortex and hippocampus [37–39]. A mechanism that accounts for the increase in SOD1 and SOD3 activity is their stabilization by zinc [40]. Also, the antioxidant effect of zinc might be due to the induction of metallothioneins, which are involved in the homeostasis of zinc and ROS [41]. Our results also show that the therapeutic administration of selenium maintains the level of enzymatic activity and transcription of SOD2 in the early phase, SOD1 in the late phase, and SOD3 in the complete period of the study post-CCAO. These three enzymes could have provided an effect of resistance/tolerance to ischemia in the early and late phases of cerebral hypoxia-ischemia, as reported in transgenic mice [42–45]. Furthermore, the effects of SOD on preventing the disruption of the blood-brain barrier [46], decreasing karyorrhexis, and attenuating the activation of NF-*κ*B [47] might also explain the neuroprotection induced by the combined treatment with zinc and selenium. Results in *sod1* [48], *sod2*, and *sod3* knockout models [37, 49, 50] also confirmed the neuroprotective effect of SOD. Accordingly, the deficiency of SOD enzymatic activity in the late phase of ischemia has been associated with increases in the size of the infarction, the release of cytochrome c, and the production of mitochondrial superoxide radicals [51]. Of the three SOD isoforms, SOD2 is thought to be the primary contributor to the protective effect in both transient and permanent occlusion [52, 53]. Our results support this proposal.

We found that the combined treatment with zinc and selenium also causes upregulation of *Gpx4* in the early and late phases of CCAO. This result suggests that GPx4 also contributes to the neuroprotective effect of zinc and selenium, removing peroxides from cell membranes and macromolecules such as lipids, proteins, and DNA [54, 55]. Another mechanism of neuroprotection by GPx4 is to prevent apoptosis, counteracting the activity of lipoxygenase (LOX) [56] and promoting survival and proliferation [57]. In agreement with the antiapoptotic effect, *Gpx* knockout mice develop an increased volume of myocardial infarction in a hypoxia-ischemia event [58]. Furthermore, selenium can inhibit TRPM2 and TRPV1 receptors (activated by increasing H_2_O_2_), thus preventing the entry of calcium into the cell that is known to detonate oxidative stress and inflammation [59].

Our results show that CCAO upregulated the mRNA for *Cxcl12/Cxcr4* and *Cxcl13/Cxcr5* without modifying their protein levels, although it decreased CXCL13 protein levels. In contrast with our transient CCAO model for 10 min, the permanent occlusion of the middle cerebral artery (MCAO) increases CCL2, CXCL2, and CXCL13 levels after 2 days, gradually decreasing after 7 days of MCAO [60, 61]. Therefore, the transient effect of hypoxia-ischemia might be insufficient to alter the protein levels of chemokines and receptors in the time points we have studied. Moreover, the lack of the effect in protein levels can be explained by posttranscriptional regulation of miRNAs [62, 63] or a posttranslational regulation at the level of degradation after receptor-ligand desensitization [64]. In this latter case, those chemokines could have exerted their function before their degradation. Then, the upregulated *Ccl2/Ccr2* by the combined zinc and selenium administration in the late phase of hypoxia-ischemia might be neuroprotective because they are known to decrease cell death and improve memory [65]. Furthermore, CCL2 also stimulates the migration of neuronal precursor cells to the damaged area [66]. We have previously reported that high levels of CCL2 by a subacute prophylactic administration of zinc are associated with a preconditioning process [5]. However, the combined treatment with zinc and selenium did not maintain the preconditioning effect of zinc but exerted the therapeutic effect of selenium in the late phase. This effect is reflected by a decrease in cell death and recovery of long-term memory.

CXCL12 and CXCL13 have been associated with a deleterious role during cerebral ischemia [67] attracting lymphocytes [68]. Nevertheless, CXCL12 and CXL13 can also attract neuronal precursor cells mainly through interaction with CXCR4 or CXCR5, respectively [69–72]. CXCL12 and CXCL13 promote the migration of neuroblasts from the subventricular zone in neonatal mice [73], although the main promoter of neuroblast migration is CXCL12 [74]. In this study, we found that the combined treatment with zinc and selenium upregulated *Cxcl12* and *Cxcl13* and increased CXCL13 protein levels. Therefore, these chemokines can be associated with the neuroprotective effect of the combined treatment with zinc and selenium.

The effect of selenium on memory consolidation has been shown in other models different from hypoxia-ischemia like Alzheimer's disease [75]. The facilitation of learning and improvement of cognitive development have been associated to the neuroprotective effect of zinc, which decreases free radicals produced by cerebral ischemia [76]. Our group showed similar results in the preconditioning effect of subacute zinc administration in the CCAO rat model [5]. However, the prophylactic chronic zinc administration (0.2 mg/kg of body weight/days) exerted a partial effect because there was no memory consolidation as reported previously with a higher dose of zinc [19]. In contrast, the therapeutic administration of selenium improved the long-term memory consolidation, which is consistent with the significant decrease in neuronal cell death induced by cerebral ischemia in the temporoparietal cortex.

In summary, the combination of the chronic prophylactic zinc administration with the therapeutic selenium administration exerts effective neuroprotection against transient hypoxia-ischemia. In this effect, GPx and SOD seem to be the key players in reducing oxidative stress and cell death, suggesting possible participation in neuroregeneration. The perspective of this work consists of challenging the present therapeutic strategy with a longer time of common carotid artery occlusion as it happens in humans.

## Figures and Tables

**Table 1:** List of chemokines and receptors assessed in qPCR: TaqMan probe used was obtained from Thermo Fisher Scientific Inc.

Gene	Gene name	Assay
*Ccl2*	Chemokine (C-C motif) ligand 2	Rn00580555_m1
*Ccr2*	Chemokine (C-C motif) receptor 2	Rn01637698_s1
*Cxcl12*	Chemokine (C-X-C motif) ligand 12	Rn00573260_m1
*Cxcr4*	Chemokine (C-C motif) receptor 4	Rn00573522_s1
*Cxcl13*	Chemokine (C-X-C motif) ligand 13	Rn01450028_m1
*Cxcr5*	Chemokine (C-C motif) receptor 5	Rn02132880_s1
*Nos1*	Nitric oxide synthase 1, neuronal	Rn00583793_m1
*Nos2*	Nitric oxide synthase 2, inducible	Rn00561646_m1
*Nos3*	Nitric oxide synthase 3, endothelial	Rn02132634_s1
*Sod1*	Superoxide dismutase 1, cytosolic	Rn006566938_m1
*Sod2*	Superoxide dismutase 2, mitochondrial	Rn00690588_g1
*Sod3*	Superoxide dismutase 3, extracellular	Rn00563570_m1
*Gpx4*	Glutathione peroxidase 4	Rn00820188_g1

## Data Availability

The data used to support the findings of this study are included within the article.
